# Acute Finger Ischemia in an Elderly Male without Risk Factors for Hypercoagulability

**DOI:** 10.5811/cpcem.2019.11.45224

**Published:** 2020-01-23

**Authors:** Manish Amin, Angela Torres, Phillip Aguìñiga-Navarrete, Daniel Quesada, Jason P. Jerome, Amber Jones

**Affiliations:** * Kern Medical Center, Department of Emergency Medicine, Bakersfield, California; † LAC+USC Medical Center, Department of Emergency Medicine, Los Angeles, California; ‡ Kern Medical Center, Department of Trauma Surgery, Bakersfield, California

## Abstract

Literature on ulnar artery thrombosis and acute finger ischemia is scant and usually related to underlying hypercoagulable or occlusive states, such as atrial fibrillation, thrombangiitis obliterans, vasospasm, trauma, and neurovascular compression at the root of the upper limb. An elderly hypertensive male without an underlying hypercoagulable state, and in otherwise good health, presented to our emergency department with acute multi-finger ischemia, and ulnar artery and palmar arch thromboses. Given his innocuous history, this case demonstrates the importance of maintaining acute arterial thrombosis on the differential for hand pain despite the obvious propensity toward mechanical injuries in the extremities.

## CASE PRESENTATION

A 65-year-old male with a past medical history of hypertension presented to the emergency department with sudden-onset distal fourth digit pain and paresthesia in the second through fourth digits of the right hand. His examination revealed mild distal duskiness with proximal pallor of the second through fourth digits, with mottling of his palm. Radial pulses were 2+ with weak ulnar pulses. Capillary refill was greater than two seconds. Allen’s test was positive.

**Image 1:**
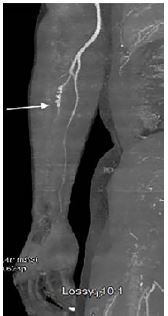
Computed tomographic angiography of the right upper extremity revealing abrupt non-opacification of the ulnar artery approximately three centimeters below the takeoff of the interosseous artery (arrow). Non-visualization of the superficial palmar arch and metacarpal arteries.

**Image 2:**
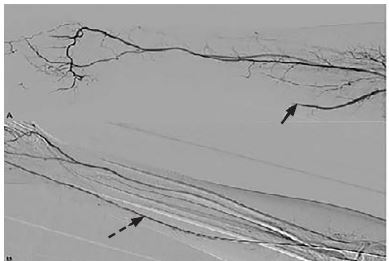
Formal angiogram of the right upper extremity (A) revealed ulnar artery flow defect pre-tissue plasminogen activator (tPA) administration (arrow). Formal angiogram of the right upper extremity (B) revealed restoration of ulnar artery flow post-tPA administration (dashed arrow).

**Image 3:**
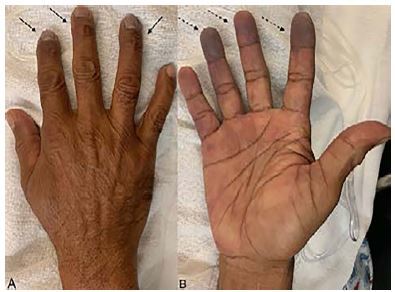
Image of the right-hand post-tissue plasminogen activator (tPA) administration. The dorsal aspect of the right hand reveals subungual pallor (A). The palmar aspect of the hand post-tPA administration (pre-tPA not pictured), revealing a significant progression of the mottling and discoloration seen on initial presentation (B).

His electrocardiogram showed normal sinus rhythm. His coagulation panel, laboratory and inpatient hypercoagulability workup was unremarkable. A computed tomographic angiogram of the right upper extremity displayed an ulnar-artery filling defect (Image 1). A heparin drip was initiated in consultation with vascular surgery. A formal angiogram of his upper extremity revealed a similar filling defect (Image 2A), at which point intra-arterial tissue plasminogen activator (tPA) was initiated. On hospital day two, the patient had a return of a strong ulnar pulse and improved perfusion to the affected digits (Images 2B, 3A, and 3B).

## DISCUSSION

Common etiologies of acute finger ischemia include but are not limited to hypercoagulable states, atrial fibrillation, thrombangiitis obliterans, vasospasm, trauma, and neurovascular compression at the root of the upper limb.^1^ This case demonstrates the importance of physical exam maneuvers such as neurovascular testing, assessment of capillary refill and Allen’s test in diagnosing critical limb ischemia where a history of risk factors for arterial thrombi is absent. The management of limb ischemia includes initiating anti-platelet therapy and heparin to prevent further thrombosis.^5^ Restoration of blood flow can be achieved by intra-arterial thrombolytic infusions in conjunction with interventional radiology, surgical revascularization, or thrombectomy.^5^ A retrospective, single-center study revealed a trend toward increased amputation-free survival in patients who underwent thrombolysis after acute finger ischemia; however, the study lacked power.^2^^”^Further studies are needed to delineate treatment guidelines for patients presenting with acute finger ischemia.

**CPC-EM Capsule**

What do we already know about this clinical entity?

Digital ischemia is an uncommon entity that occurs in patients with underlying hypercoagulable states. Treatments include anticoagulation and vascular surgery.

What is the major impact of the image(s)?

The images detail the appearance of acute finger ischemia in a patient lacking risk factors, illustrating that acute finger ischemia must be on the differential diagnosis for hand pain.

How might this improve emergency medicine practice?

In the setting of acute digit pain, even with a relatively benign physical exam, a high index of suspicion for ischemia must be maintained.
